# Medical Civil Service Reform

**Published:** 1878-01

**Authors:** 


### MEDICAL CIVIL SERVICE REFORM.

In the day when the much vexed question of civil service reform is testing the cohesiveness of the great political organizations of this country, it is proper that medical men should enquire respecting the operation, as regards themselves, of the principles which at present control in civil appointments.

Looking at the medical corps of the military establishment, we find a body of men eminently fitted in all particulars for the discharge of their important functions. They command the high respect of the profession at large and of their associates in the "line" of the army and navy, achieving for themselves abroad a reputation based upon scientific and literary contributions of the greatest value. All this is the natural result of the operation of a very simple law. The positions of these gentlemen are obtained by success in competitive examinations. Of all applicants those best qualified, mentally, morally and physically, secure commissions. This is the system which prevails in Europe, and in accordance with which every medical man, from the hospital interne to the university professor obtains position.

By an unhappy inconsistency, the principle which was deemed essential to the welfare of the military service, and which has there produced such admirable fruit, has been utterly disregarded in the selection of medical men for the civil list. In the marine and pension services, surgeons and assistant-surgeons become such by political preferment. Not only in the offices of the general government but in those also of the states, cities and counties of our country, the appointment of physicians is determined by a system which has produced results that are often not merely deplorable, but positively disastrous.

Here in the State of Illinois, the county of Cook, and the city of Chicago, the consequences of such a policy have been sufficiently conspicuous to be seen and read of all men—consequences which are at once a reproach to the profession and a scandal to the public. There is no need of mincing words when dealing with a great outrage of this sort. Medical men have been appointed to office because of their political influence or affiliations ; or because they were relatives of those in power, or to satisfy the greedy clamor of the applicants themselves, or for purely private and personal reasons ; never solely because they were fit and qualified.

By that singular chance which occasionally evolves good from evil, and produces a rose-tree upon a dunghill, some of these offices have been held by worthy men. But what shall be said of the rest—some, the feeble and superannuated relics of a past mediocrity ; others, disappointed aspirants for private patronage, who seek to supplement their failure by pickings from the public purse ; professional incompetents and impotents ; disciples of drink, who are not ashamed of their intoxication upon the public thoroughfares ; students of medicine unprovided with a diploma, and even without that cheap-jack sort of education which too many diplomas attest ; country practitioners removed to the city solely because an enterprising and not too scrupulous relative embarked in politics—the tale cannot be fully told! It is but a few weeks since a judge upon the bench had occasion to condemn the inefficiency of one of these folk.

Meantime, the large body of medical men have been either too much absorbed in their private business to bestow any attention to this subject, or, recognizing the evil, have contented themselves with characterizing in private, the farce played before the public. Many such have signed applications for the appointment of others, from indolence, fear of offending a friend, or from ignorance of the incapacity of the applicant.

Only they who think success possible for themselves, will submit to competitive examination, when merit is to decide the issue; and only these, in a similar way, will compete in the base struggle for office, who are conscious that they possess the requisite talents—the ability to push, to beg, to fawn ; to descend to the level of the creature of the dram-shop and the bar-room politician.

There is not an office for which a medical man is required, within the territorial limits of the State, which should not be assigned to the successful competitor in examination. There is not, to-day, a competent medical officer within the same limits, who should not retain his position, so long as he faithfully and efficiently discharges its duties. He also who is worthy, should be advanced from the lower to the higher grade of duty. How much honor would a medical staff, thus constituted, reflect upon the body of the profession in this State! What a laudable ambition might be excited in the mind of the student of medicine by the contemplation of the just reward of indefatigable industry!

In the absence of any provisions to insure such a result, medical men should see to it that they put themselves right upon the record. They should not for a moment countenance the prevailing loose, and often disreputable, methods of obtaining office. A notable and honorable precedent was recently established by the Governor of Illinois, in convening the medical profession of Chicago, with a view to the nomination of a member of the State Board of Health—the nominee in this instance having received the appointment. Let the profession at large regularly nominate, by such methods as they think will secure the desired end, proper men for all medical offices, and, whether their nominations are or are not accepted, the result will be to secure a popular approval of their efforts at reform.

We are not so credulous as to suppose that the contemplated results can be accomplished in an hour. But it is one of the redeeming features of our crude aud defective system of self-government, that agitation always insures some sort of reform. In the matter of slavery, the people of the United States began by stoning the apostles of abolition, and concluded with radical and irrevocable emancipation.

And we cannot but believe that if the mass of medical men are thoroughly aroused to a sense of their high privilege and duty in this matter of medical civil-service reform, they will, in the end, accomplish the much-desired result.

